# Supplementary material for: Altered Glycolysis, Mitochondrial Biogenesis, Autophagy and Apoptosis in Peritoneal Endometriosis in Adolescents
Source: Int J Mol Sci. 2024 Apr 11;25(8):4238. doi: 10.3390/ijms25084238 (PMC11050237; doi:10.3390/ijms25084238)
Supplement: Supplementary file 1 [file ijms-25-04238-s001.zip › ijms-2915801-supplementary.pdf]

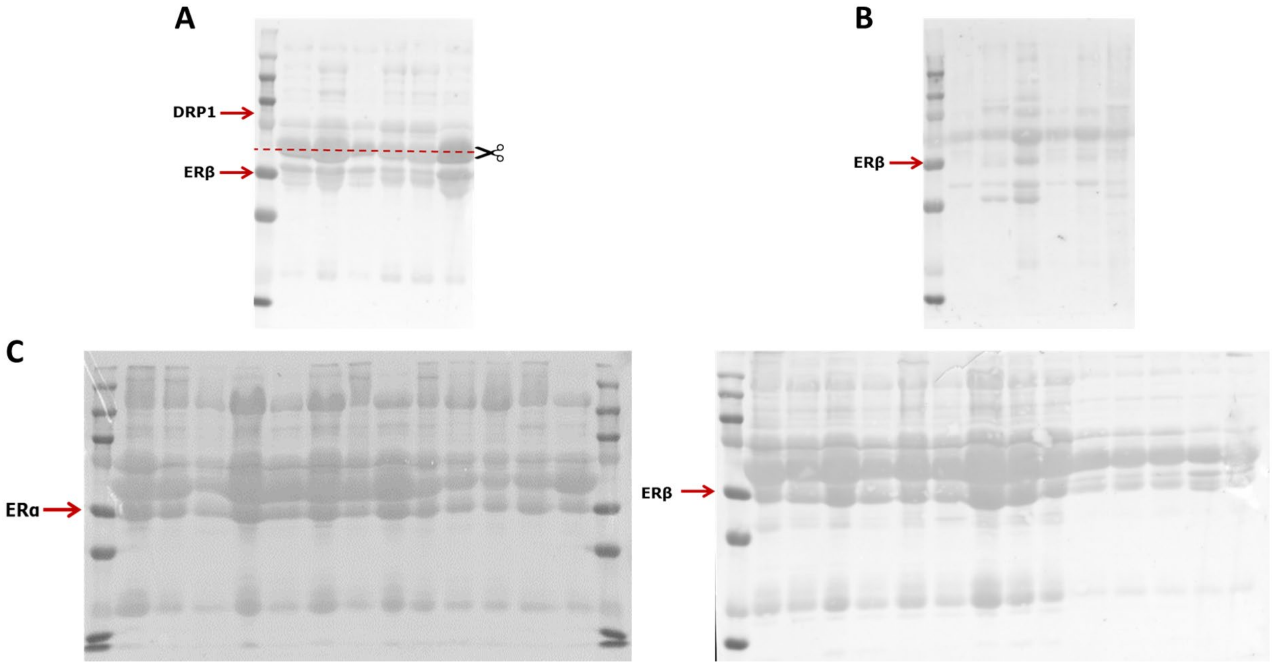

Figure S.1. Ponceau staining images for ERβ levels in exosomes from blood (**A**), peritoneal fluid **(B)** and ERα and ERβ levels in tissues (**C**).

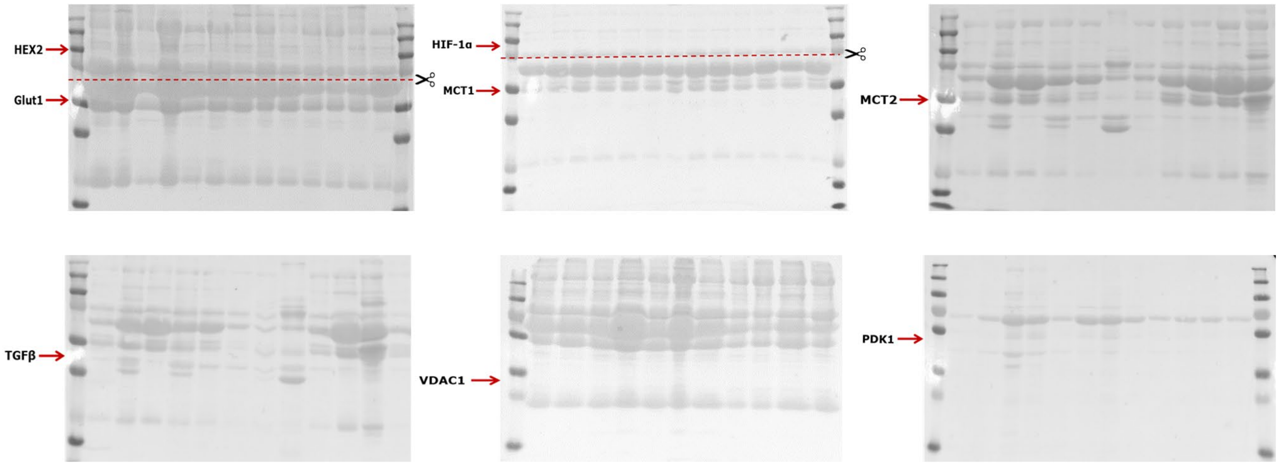

Figure S.2. Ponceau staining images for Glut1, Hex2, Hif-1α, MCT1, MCT2, TGFβ, VDAC1, PDK1 markers in tissue biopsies.

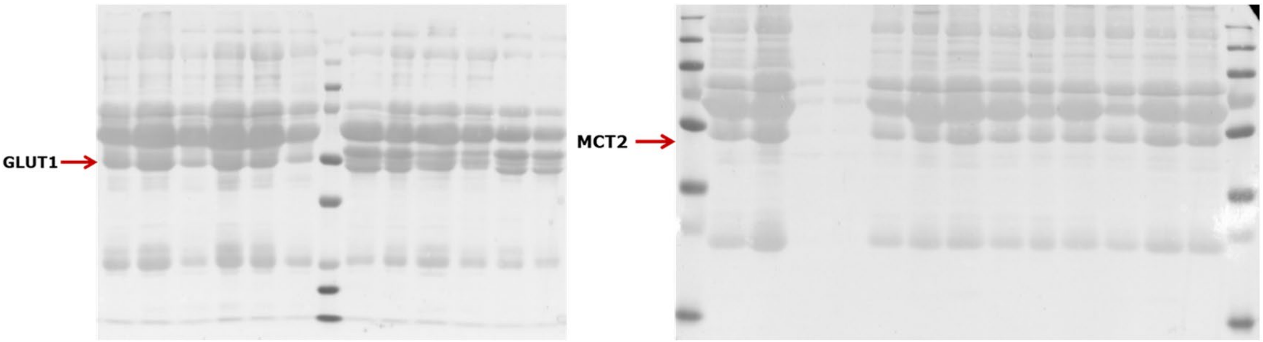

Figure S.3. Ponceau staining images for Glut1 and MCT2 in blood exosomes.

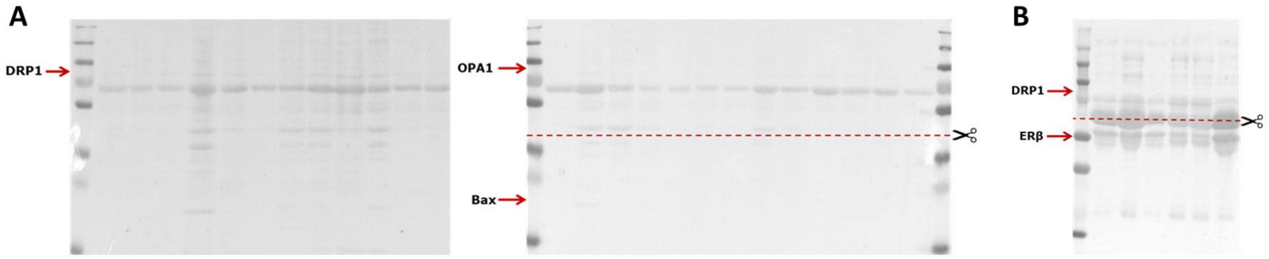

Figure S.4. Ponceau staining images for DRP1, OPA1 levels in tissues (**A**) and DRP1 level in blood exosomes (**B**).

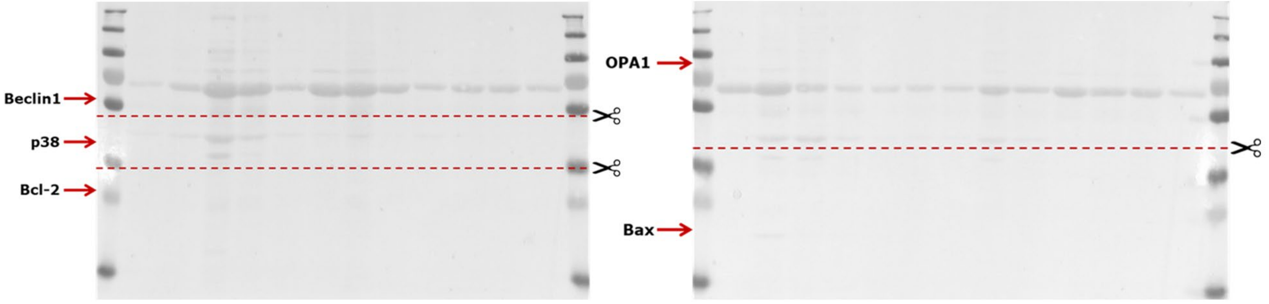

Figure S.5. Ponceau staining images for Bcl-2, p38, Becklin1 (**A**) and Bax, Opa1 (**B**) levels in tissues.

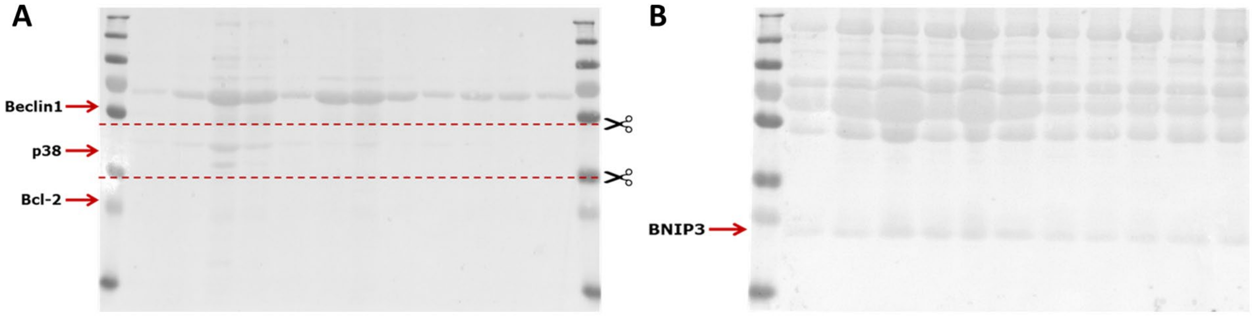

Figure S.6. Ponceau staining image for p38 and Beclin1 levels in tissues (**A**) and BNIP3 level in blood exosomes (**B**).

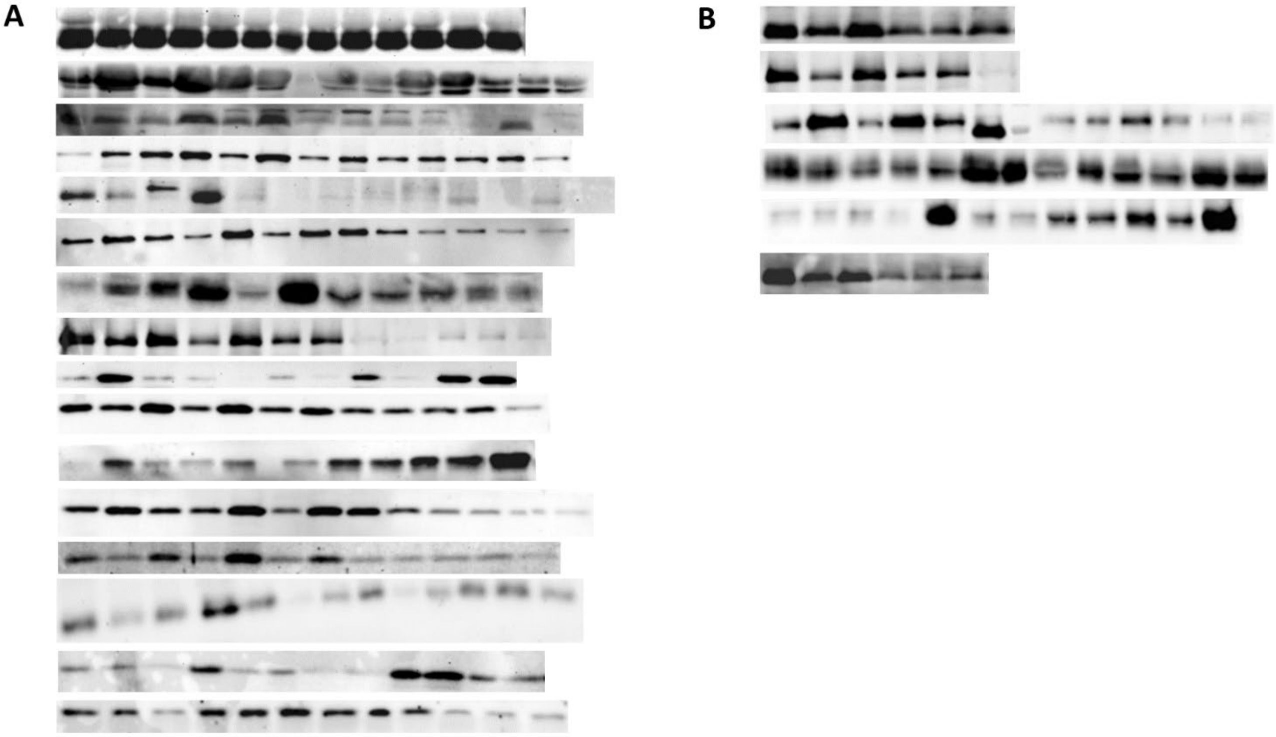

Figure S. 7. Original images obtained by Western blot for tissues (**A**) and exosomes (**B**). For description see Figures 1-6.
